# Structural basis of the XPB helicase–Bax1 nuclease complex interacting with the repair bubble DNA

**DOI:** 10.1093/nar/gkaa801

**Published:** 2020-09-28

**Authors:** Feng He, Kevin DuPrez, Eduardo Hilario, Zhenhang Chen, Li Fan

**Affiliations:** Department of Biochemistry, University of California, Riverside, CA 92521, USA; Department of Biochemistry, University of California, Riverside, CA 92521, USA; Department of Biochemistry, University of California, Riverside, CA 92521, USA; Department of Biochemistry, University of California, Riverside, CA 92521, USA; Department of Biochemistry, University of California, Riverside, CA 92521, USA

## Abstract

Nucleotide excision repair (NER) removes various DNA lesions caused by UV light and chemical carcinogens. The DNA helicase XPB plays a key role in DNA opening and coordinating damage incision by nucleases during NER, but the underlying mechanisms remain unclear. Here, we report crystal structures of XPB from *Sulfurisphaera tokodaii* (St) bound to the nuclease Bax1 and their complex with a bubble DNA having one arm unwound in the crystal. StXPB and Bax1 together spirally encircle 10 base pairs of duplex DNA at the double-/single-stranded (ds–ss) junction. Furthermore, StXPB has its ThM motif intruding between the two DNA strands and gripping the 3′-overhang while Bax1 interacts with the 5′-overhang. This ternary complex likely reflects the state of repair bubble extension by the XPB and nuclease machine. ATP binding and hydrolysis by StXPB could lead to a spiral translocation along dsDNA and DNA strand separation by the ThM motif, revealing an unconventional DNA unwinding mechanism. Interestingly, the DNA is kept away from the nuclease domain of Bax1, potentially preventing DNA incision by Bax1 during repair bubble extension.

## INTRODUCTION

Transcription and DNA repair are two essential biological processes. As the largest subunit of the transcription factor TFIIH complex (1), XPB is required for promoter melting in transcription and unwinding damaged DNA in nucleotide excision repair (2–4). Mutations in XPB are associated with xeroderma pigmentosum (XP), trichothiodystrophy (TTD) and Cockayne syndrome (CS) symptoms with developmental disorders or increased frequency of skin cancer (5,6). XPB is a superfamily 2 (SF2) DNA helicase conserved from archaea to human (1,7–10). In transcription, XPB binds dsDNA downstream from the promoter opening location (11) and has been proposed to function as a “molecular wrench" (12) or dsDNA translocase (13–15). In the general genomic NER pathway, DNA lesions are first recognized by the XPC-HR23B complex (16), which directly recruits the TFIIH complex for DNA unwinding (17–21). TFIIH uses its helicase subunits XPB and XPD to generate the repair bubble. XPB likely initiates DNA unwinding at the lesion (2,22) since XPD is a conventional SF2 helicase and requires a ssDNA extension to start unwinding (23–26). Other NER factors including XPA and replication protein A (RPA) are required to facilitate the assembly of the preincision complex (2). After the DNA lesion is verified by TFIIH, the ERCC1–XPF complex and XPG nucleases incise the damaged strand at the 5′ and 3′ to the lesion, respectively, to remove a damage-containing fragment of about 25–30 nucleotides (27–29). The gap is finally filled by the DNA replication machinery (2). However, it is unclear how XPB recognizes the DNA substrate and initiates unwinding in NER. Structural analysis on crystal structures of *Archaeoglobus fulgidus* XPB (AfXPB) (7) and StXPB (30) suggested that domain rotation in XPB might generate a supertwist in DNA at the lesion, leading to the initial unwinding, consistent with the recent cryo-EM structure of XPA and the TFIIH core bound to a forked DNA substrate showing that human XPB acts as a translocase by binding to the dsDNA region ahead of the fork during DNA repair (31). In archaea, due to the lack of the TFIIH-like complex, XPB is in complex with Bax1, an XPG-like nuclease, to function as a helicase-nuclease machine for DNA unwinding and incision (32–34). We recently reported the crystal structures (35) of the XPB-Bax1 complex from both *Archaeogloubus fulgidus* and *Sulfurisphaera (previously named Sulfolobus*) *tokodaii*. These structures reveal that the XPB-Bax1 complex is a dynamic machinery which can adapt different conformations for protein-protein and protein-substrate interactions.

Here we determined the crystal structures of the StXPB–Bax1^ΔC^ (a truncated Bax1 without the C-terminal domain, which is absent in many archaeal Bax1 homologs (35)) complex and the StXPB–Bax1^ΔC^ heterodimer associated with a bubble DNA substrate, which has one dsDNA arm unwound in the crystal to become a forked DNA. StXPB in the DNA-free heterodimeric structure contains a phosphate ion in its ATP-binding site, possibly mimicking the state of StXPB after ATP hydrolysis (ADP + phosphate). Structural and mutational analyses reveal that the conserved RED and ThM motifs play key roles in DNA interactions and XPB activities, consistent with previous results on both human and archaeal XPB (7,36). These results provide new insights into the molecular mechanisms of XPB-mediated DNA repair bubble formation in archaeal and eukaryotic NER.

## MATERIALS AND METHODS

### Cloning, expression and purification of StXPB and StXPB-Bax1^ΔC^

The DNA encoding StXPB (residues 2–439) was cloned into a modified pET28a vector with an N-terminal His_8_-tag followed by a PreScission protease cleavage site, while the DNA encoding a truncated StBax1^ΔC^ (residues 2–373) was cloned into the pET15b vector by PCR. Purification of StXPB was described previously (30). StXPB and Bax1^ΔC^ were co-expressed in *Escherichia coli* Rosetta (DE3) pLysS cells (Invitrogen). After induction for 18 h with 0.2 mM IPTG at 28°C, the cells were harvested by centrifugation and the pellets were resuspended in lysis buffer containing 50 mM Tris–Cl pH 7.5, 500 mM NaCl, 10% glycerol. The cells were then lysed by sonication and the cell debris was removed by centrifugation. The supernatant was purified by affinity chromatography using Ni-NTA resin (Thermo Scientific). PreScission protease was then added to remove the His_8_-tag. The protein complex was further purified by HiTrap SP FF ion-exchange chromatography (GE). The purification was completed by gel-filtration chromatography (Superdex 200, 16/60, GE) in 25 mM Tris–Cl pH 7.5, 200 mM NaCl or 25 mM HEPES pH 7.5, 200 mM NaCl (for crystallization). The purified protein samples were concentrated and stored at −80°C. All the variants of StXPB were expressed and purified following similar procedures.

### Crystallization and structure determination

Crystals of the StXPB-Bax1^ΔC^ complex were prepared from 200 mM NH_4_-citrate pH 7.5, 8% PEG3350 by the sitting-drop vapor diffusion at room temperature. Synthesized DNA oligos are used as additives in the drop to promote crystal formation. Crystals grew as plates to maximal size within 1 week. Crystals were gradually transferred into a harvesting solution made of mother liquor supplemented with 26% ethylene glycol, followed by flash-freezing in liquid nitrogen for shipment to synchrotron facilities. X-ray diffraction datasets for StXPB-Bax1^ΔC^ complex were collected at beamline 5.0.1 at the Advanced Light Source, Lawrence Berkeley National Laboratory, and the diffraction data were indexed, integrated, and scaled using the HKL3000 program (37). The structure was solved by molecular replacement using Phaser (38), with individual domains of the StXPB–Bax1 structure (PDB entry: 6P4O) (35) as search models. Protein structure refinement was carried out with the REFMAC5 (39).

The StXPB-Bax1^ΔC^–DNA complex was crystallized by sitting-drop vapor diffusion at room temperature. The StXPB–Bax1^ΔC^ complex was mixed with the bubble-6 DNA at a protein:DNA ratio of 1:1.2, followed by incubation for 40 min at room temperature. The protein–DNA co-crystals typically grew in a reservoir solution consisting of 50 mM MES pH 5.3, 10 mM MgCl_2_, 26% 2-methyl-2,4-pentanediol (MPD). The quality of crystals was improved by micro-seeding. Crystals grew as plates to maximal size in 2 weeks. Crystals were transferred into a harvesting solution containing 50 mM MES pH 5.3, 10 mM MgCl_2_ and 28% MPD, followed by flash-freezing in liquid nitrogen. More than 50 different DNA substrates (including ssDNA and dsDNA of different sizes, dsDNA with different overhangs, forked DNA with different arms, and dsDNA with different bubble sizes, etc.) were tested in co-crystallization trials and well-diffracting co-crystals were obtained only with the bubble-6 DNA. The dataset for the XPB-Bax1^ΔC^-DNA complex was collected on the 24-ID-C NE-CAT beamline at the Advanced Photon Source, Argonne National Laboratory, and the diffraction data were indexed and integrated using iMOSFLM (40), then scaled and merged with SCALA (41). The structure was solved by molecular replacement with Phaser (38) using individual domains of the StXPB-Bax1^ΔC^ structure as search models. Positive density appearing in the difference map was identified as DNA, which was manually built into the density and improved in Coot (42), refinement was performed using the PHENIX software package (43). All the structural figures were prepared with PyMOL (www.pymol.org).

### Cloning, expression and purification of human XPB-p52-p8 trimer

The DNA encoding the full-length human XPB was cloned into a modified Bac-to-Bac vector with an N-terminal His_6_-tag followed by a PreScission protease cleavage site. The DNA encoding the full-length human p52 and p8 were cloned into MacroBac 438A vector, and then p52 and p8 were combined into a single vector via restriction digestion and ligation-independent cloning (44). The recombinant baculovirus expressing XPB or p52/p8 was generated using standard protocols. High Five insect cells were co-infected with these two recombinant baculoviruses. The cells were harvested after 70 hours by centrifugation. The pellets were resuspended in lysis buffer containing 50 mM Tris–Cl pH 7.0, 500 mM NaCl, 10% glycerol, 1 mM PMSF. The cells were then lysed by sonication, and the debris was removed by ultracentrifugation. The supernatant was mixed with Ni-NTA resin and rocked for 1 h at 4°C before elution with 400 mM imidazole. PreScission protease was then added to remove the His_6_-tag. The proteins were further purified by ion-exchange chromatography (SP-FF, GE) and gel-filtration chromatography (Superdex 200, 16/60, GE). The purified protein samples were concentrated in 25 mM Tris–Cl pH 7.5, 200 mM NaCl, 5% glycerol, 2 mM DTT, and stored at –80°C.

### Cloning, expression and purification of human XPA

The DNA encoding the full-length human XPA was cloned into a modified pET28a vector with a cleavable N-terminal His_6_-SUMO tag for expression in *E. coli* Rosetta (DE3) pLysS cells (Invitrogen). After induction for 18 h with 0.2 mM IPTG at 22°C, the cells were harvested by centrifugation and the pellets were resuspended in lysis buffer containing 50 mM Tris–Cl pH 7.5, 500 mM NaCl, 10% glycerol. The cells were then lysed by sonication and the cell debris was removed by centrifugation. The supernatant was purified by Ni-NTA affinity chromatography and SUMO protease was then added to remove the His_6_-SUMO tag. XPA was further purified with the Heparin (GE) and Superdex 200 (16/60, GE) columns. The purified XPA protein samples were concentrated in 25 mM Tris–Cl pH 7.5, 200 mM NaCl, 5% glycerol, 2 mM DTT, and stored at –80°C.

### Electrophoretic mobility shift assay

For the forked DNA substrate, unless otherwise indicated, 0.8 μM DNA was incubated with 0.4, 0.8 μM StXPB, StXPB–Bax1^ΔC^, human XPB-p52-p8, human XPA or both XPB-p52-p8 and XPA (each at 0.4 or 0.8 μM) in 10 μl binding buffer at room temperature for 40 min. For the bubble DNA substrates, unless otherwise indicated, 0.3 μM DNA was incubated with 0.3, 0.6 μM StXPB in 10 μl binding buffer at room temperature for 40 min. The binding buffer consists of 25 mM Tris–Cl pH 7.5, 100 mM NaCl, 5% glycerol, 1 mM DTT. All samples were loaded and resolved in 4% TBE native gel under 100 V for 0.5 h. The gels were then stained by ethidium bromide and visualized under UV light.

### ATPase activity assay

ATPase reactions were carried out in a 20 μl reaction buffer (50 mM Tris–Cl pH 7.5, 100 mM KCl, 5 mM MgCl_2_, 1 mM DTT) with 1 mM ATP. 1 μM StXPB or StXPB–Bax1 WT and mutants were assayed in the absence or presence of 1 μM DNA substrate in a 50°C water bath for 10 min. The concentration of liberated phosphate from hydrolyzed nucleotides was detected as previously described (35). The absorbance of reactions with nucleotide alone was subtracted from protein reactions to account for ATP auto-hydrolysis.

## RESULTS

### Overall structure of the XPB-Bax1-DNA ternary complex

**Figure 1.**
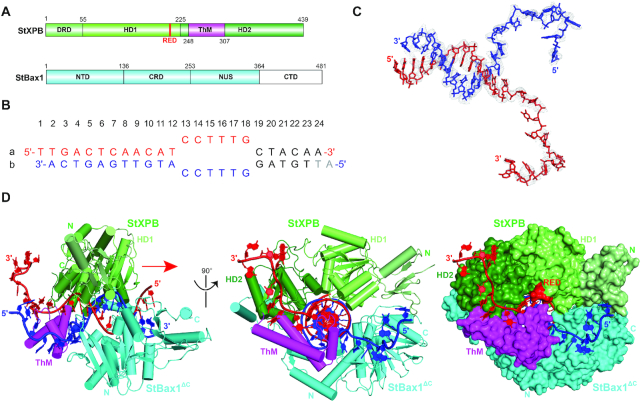
Structure of the StXPB–Bax1^ΔC^-forked DNA complex. (**A**) Diagrams of domain arrangements in StXPB and StBax1. Domains are presented as boxes in different colors with labels: DRD (damage recognition domain), HD1 (helicase domain 1), HD2 (helicase domain 2) and ThM (thumb-like) domains of StXPB are colored in palegreen, lime, forest green and magenta; StBax1 are colored in cyan with the truncated C-terminal domain in white. (**B**) Sequence of the bubble-6 DNA substrate used for crystallization. The two DNA strands (strand a and b) are colored in red and blue, respectively. Unwound bases in the crystal are in black and missing bases are in gray. (**C**) The electron density (*F*_o_ – *F*_c_) map for the forked DNA is contoured at 2σ level in gray. (**D**) Left: Orthogonal views of the StXPB–Bax1^ΔC^-forked DNA complex structure in cylindrical representation. The red arrow indicates the direction for StXPB to move along the red DNA strand for dsDNA unwinding. Right: the StXPB–Bax1^ΔC^-forked DNA complex structure with both proteins presented in surfaces and DNA in ribbons. Protein domains/motifs are colored as in (A).

**Figure 2.**
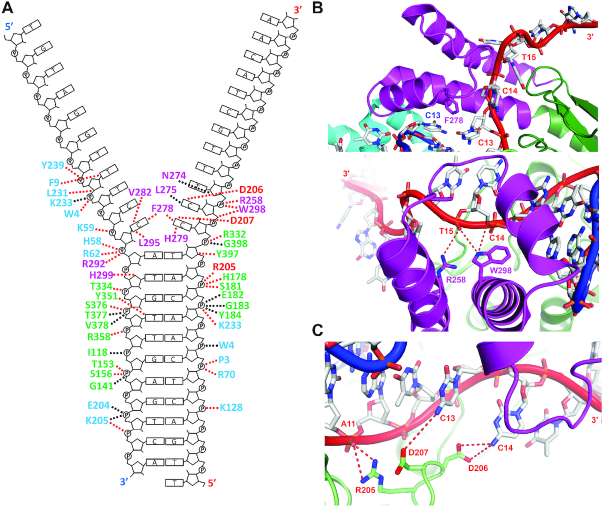
Interactions of the StXPB-Bax1 heterodimer with the forked DNA substrate. (**A**) Diagram of DNA–protein interactions. Residues directly contacting DNA are shown with interactions to DNA highlighted by dashed lines: red lines for interactions from residue side-chain and black lines for interactions from peptide backbone. Residues from Bax1 are in cyan, residues from the HD1/HD2 and the ThM of XPB are in green and magenta, respectively. The RED motif residues (R205, D206 and D207) are highlighted by red labels. (**B**) The ThM motif intrudes between the two ssDNA arms (DNA backbones are in red and blue ribbons, respectively) and grips the 3′-overhang (red ribbon) with residue F278 stacking with C13_a_–C13_b_ (Top) and residue R258 interacting with T15_a_ while W298 interacting with both C14_a_ and T15_a_ (bottom). (**C**) The RED motif interacts with the junction with residue R205 forming hydrogen bonds with A11_a_ and residues D206 and D207 stabilizing the unpaired bases of C14_a_ and C13_a_, respectively. DNA and protein backbones are displayed as ribbons with the same colors as in Figure 1D. Nucleotides and key amino acid residues are shown in sticks with oxygen atoms in red and nitrogen atoms in blue.

Extensive trials on crystallizing the full-length StXPB–Bax1 complex with DNA did not achieve diffracting quality crystals, so we engineered a truncated StBax1 by removing its C-terminal domain (Figure 1A), which is absent in many archaeal Bax1 orthologues. The crystal structure of the StXPB–Bax1^ΔC^–DNA ternary complex was determined at 3.55 Å resolution (see Supplementary Table S1 for statistics of data collection and structure refinement). The StXPB–Bax1^ΔC^ complex interacts with DNA in the same way as the StXPB-Bax1 complex does (Supplementary Figure S1). The DNA substrate used for the co-crystallization is a 24 base-pair (bp) DNA duplex containing a 6-nucleotide unpaired region (hereafter, bubble-6 DNA, Figure 1B). Surprisingly, the 6-bp short arm of the bubble-6 DNA was unwound in the crystal (Figure 1C), which is consistent with our previous observation that binding of XPB to DNA induces changes in DNA electrochemical properties even in the absence of ATP (30). The dsDNA region retains the B form while the two ssDNA tails are bent and split apart by XPB and Bax1^ΔC^, respectively (Figure 1D). Bax1^ΔC^ contains three domains (Figure 1A and D): the N-terminal domain consisting of two helix-bundles (NTD), the central Cas2-like domain (CRD) (35) and the nuclease domain (NUS). The DNA-bound StXPB-Bax1^ΔC^ heterodimer spirally encircles the DNA substrate by the HD1/HD2/ThM of XPB and the NTD/CRD of Bax1 (Figure 1D), forming a tunnel for 10-bp DNA duplex binding with XPB closer to the fork (Figure 2A). Furthermore, the ThM motif of XPB intrudes between the two ssDNA tails like a wedge with the 3′-overhang extending through the channel formed by the HD2/ThM of XPB (Figures 1D and 2A and B) and the 5′-overhang extending into the space between two N-terminal β-hairpins of Bax1^ΔC^ (Figures 1D and 2A). These observations are consistent with the 3′–5′ helicase polarity of archaeal XPB (7) (moving along the 3′-overhang strand toward the fork junction) and the nuclease activity of Bax1 on the DNA substrate containing a 5′-overhang (33) *in vitro*. Neither XPB nor Bax1^ΔC^ interacts with the remaining nucleotides of the two ssDNA tails further away from the fork, leading to poor electron density for this portion of the DNA.

### Interactions between the XPB–Bax1^ΔC^ heterodimer and the forked DNA

Close examination of the interface between the StXPB–Bax1^ΔC^ heterodimer and the forked DNA reveals how XPB and Bax1 interact with DNA at the ds–ss DNA junction. XPB makes extensive contacts to dsDNA (base pairs 6–12) immediately adjacent to the junction, the first mismatching base pair C13_a_–C13_b_, and the next two unpaired nucleotides C14_a_ and T15_a_ on the 3′-overhang (Figure 2 and Supplementary Figure S2). The interactions of XPB with the ds-ss DNA junction region are mainly mediated by residues from the RED and ThM motifs (Figure 2A), two unique and important motifs among XPB homologues (7,36). The ThM motif grips the 3′-overhang like a claw (Figure 2B). Residues N274, L275, F278, H279, V282, L295 intrude between the two ssDNA tails and interact with C13_a_-C13_b_, C14_a_ and T15_a_, and the aromatic side chain of residue F278 approaches and stacks with the mismatched C13_a_–C13_b_ (Figure 2A and B), very similar to the F633 (45) or Y621 (46) at the separation pin of UvrD. Residue R205 (of the RED motif) forms hydrogen bonds with the phosphate of nucleotide A11_a_ and residue D206 (of the RED motif) stabilizes the unpaired base of C14_a_ while residue D207 (of the RED motif) interacts with the mismatched base of C13_a_ (Figure 2A and C). The side chains of R258 and W298 (of the ThM motif) interact with the phosphate backbone of T15_a_ and W298 also interacts with the phosphate backbone of nucleotide C14_a_ (Figure 2A and B). The DNA duplex immediately adjacent to the fork sits in the upper section of the groove formed between the two RecA-like motor (HD1, HD2) domains (Figure 1D). The bottom of the same groove is the site for ATP binding and hydrolysis (Supplementary Figure S3). Therefore, conformational changes induced by ATP binding and hydrolysis likely push StXPB to move along the dsDNA. When XPB translocates along the dsDNA ahead of the fork, the ThM motif grips the 3′ overhang tail and the tip of the ThM motif, particularly residue F278, functions as a wedge to break the base pairs along the way. Collectively, these interactions allow StXPB to function as a dsDNA translocase with 3′-5′ helicase activity. In the ternary complex, Bax1^ΔC^ interacts with the unpaired 5′-overhang nucleotides C13_b_, C15_b_, T16_b_ and stabilizes the strand separation, likely enhancing the DNA unwinding by XPB. In addition, Bax1^ΔC^ has some contacts with the dsDNA (base pairs 3–8, 10, 12) next to XPB (Figure 2A) and extends the protein-dsDNA interactions, possibly increasing the processivity of DNA unwinding by XPB. However, the nuclease domain of Bax1^ΔC^ does not interact with DNA at all, suggesting that the nuclease activity is inhibited when the repair bubble is being created and extended by XPB helicase during DNA repair. This is consistent with the previous study showing that XPB inhibits the endonuclease activity of Bax1(32).

**Figure 3.**
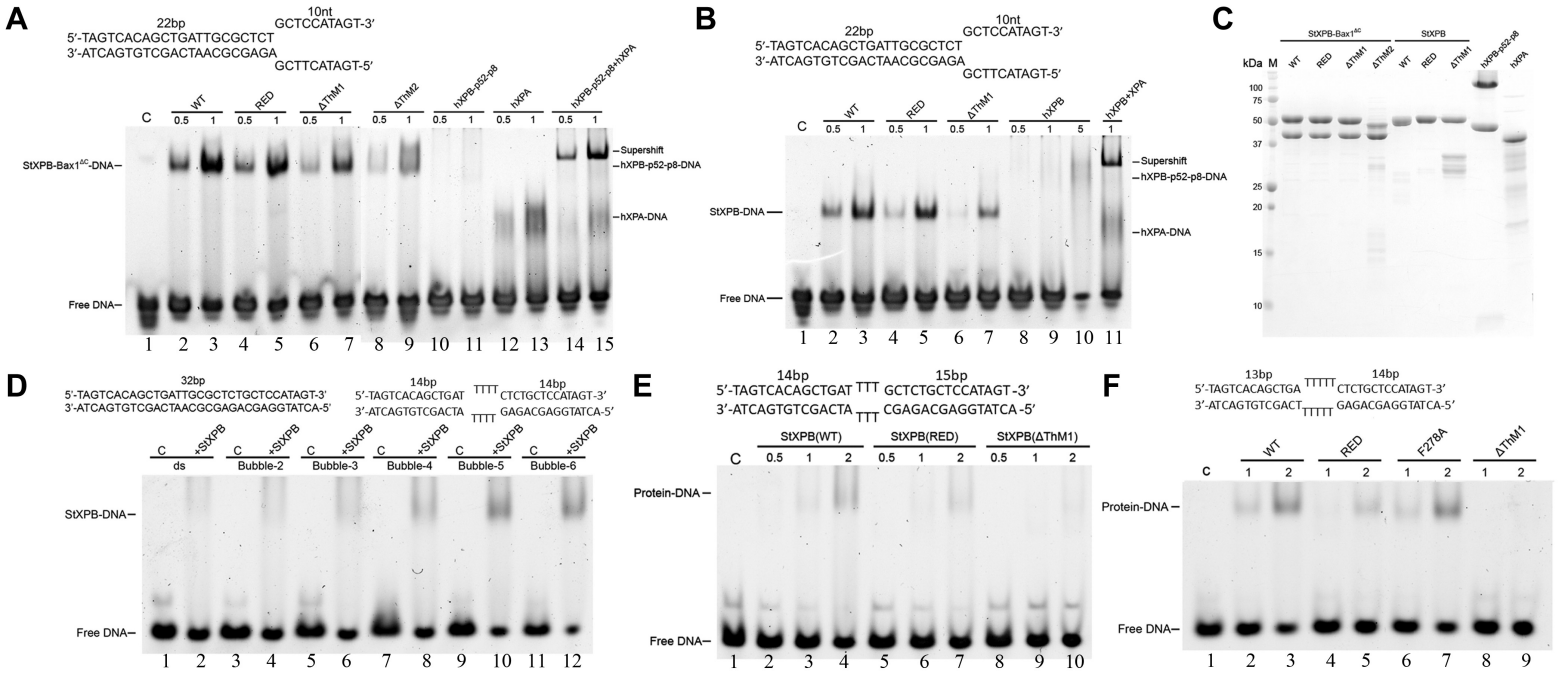
The RED and ThM motifs of XPB are important for DNA binding. EMSA analysis on the interactions of a forked DNA substrate (sequence shown above the gel) with increasing concentrations of the StXPB–Bax1^ΔC^ complex containing StXPB variants (**A**), and StXPB variants compared with human XPB-p52-p8 and XPA (**B**). (**C**) SDS-PAGE results of protein samples used for EMSA. WT: the StXPB-Bax1^ΔC^ heterodimer or StXPB, RED: StXPB mutant with R205A/D206A/D207A substitutions, ΔThM1: StXPB mutant with deletion of residues 270 to 280, ΔThM2: StXPB mutant with deletion of residues 258 to 299. hXPB: human XPB-p52-p8 complex, hXPA: human XPA. (**D**) EMSA analysis on the interactions of StXPB with dsDNA substrate (sequence shown above the gel) and dsDNA with 2-nt mismatch (bubble-2), 3-nt mismatch (bubble-3), 4-nt mismatch (bubble-4, sequence shown above the gel), 5-nt mismatch (bubble-5), and 6-nt mismatch (bubble-6). (**E**) EMSA analysis on the interactions of StXPB variants with bubble-3 DNA. (**F**) EMSA analysis on the interactions of StXPB variants with bubble-5 DNA. Each EMSA gel is a representative of the same EMSA experiment repeated at least twice. C: control reaction of the DNA substrate alone. The molar ratios of protein to DNA are indicated on the top of the gels. Lane numbers are marked at the bottom of the gels.

To confirm the importance of the RED and ThM motifs for StXPB activities, variants of StXPB containing substitutional mutations R205A/D206A/D207A in the RED motif or deletion of residues 270–280 (ΔThM1) and residues 258–299 (ΔThM2) in the ThM motif were prepared, and the effects of these mutations on DNA binding of the StXPB-Bax1 complex or StXPB alone were analyzed (Figure 3). Results from EMSA assay revealed that Bax1 enhances the affinity of StXPB binding to the forked DNA (comparing lane 2–3 in Figure 3A to lane 2–3 in Figure 3B). As indicated by the ternary complex structure, mutations of the RED or ThM motif could disrupt the interactions of the heterodimer or StXPB with the forked DNA (Figure 3). Substitutions of three charged residues in the RED motif with alanine reduced the affinity of StXPB or the StXPB-Bax1 complex with the forked DNA substrate (compare lane 4 with lane 2 in Figure 3A and B) while deletion (ΔThM1) of the tip of the ThM motif reduced the affinity even further (lane 6 in Figure 3A and B). Furthermore, the heterodimer containing the deletion mutant ΔThM2 (this mutant is so unstable that we could not purify it without its partner Bax1) mimicking the short ThM motif of the human XPB has the lowest DNA binding affinity and forms unstable protein-DNA complexes (lanes 8–9 in Figure 3A). For comparison, we also analyzed the DNA-binding affinity of human XPB expressed in insect cell culture by baculovirus expression system together with p52 and p8 (when being expressed alone, human XPB is insoluble). Human XPB (p52-p8) formed even weaker and unstable complexes with the forked DNA substrate (the smear bands in lanes 10–11 in Figure 3A and lanes 8–10 in Figure 3B). However, XPA helps human XPB (p52-p8) to form a stable complex with the forked DNA substrate (lanes 14–15 in Figure 3A and lane 11 in Figure 3B), consistent with the recent cryo-EM structure showing XPA hooked human XPB at the fork of the DNA repair bubble (31). XPA itself also forms unstable complexes with the forked DNA substrate (lanes 12–13 in Figure 3A). These results together suggest that the longer ThM motif of archaeal XPB (compared to human XPB) may replace the need for XPA in archaeal NER since no XPA homologs have been identified so far in archaea.

### XPB conformational changes induced by DNA binding

**Figure 4.**
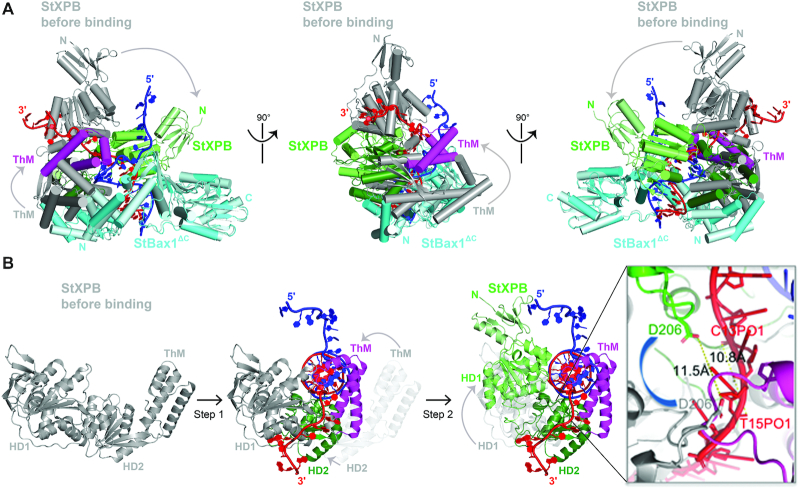
DNA interactions induce conformational changes in StXPB. (**A**) Comparison of the StXPB–Bax1^ΔC^ heterodimer with the DNA bound ternary complex by superimposing the two StBax1^ΔC^ molecules over each other in three different views. The DNA-bound StXPB-Bax1^ΔC^ ternary structure is shown in ribbons with the same colors as in Figure 1D. In the DNA-free StXPB–Bax1^ΔC^ structure, StXPB is colored in gray and StBax1 is colored in palecyan. The curved arrows indicate the movements of the N-terminal half and ThM of StXPB from DNA free state (gray) to the DNA-bound state (green). (**B**) Structure-based molecular mechanism for StXPB to unwind a forked DNA in two steps. Step one: DNA-free StXPB (and Bax1^Δ^, omitted for simplicity) binds to a forked DNA. DNA sits at the upper section of the groove between HD1 and HD2 of StXPB to allow the ThM motif to clamp down (curved light-gray arrow) at the fork; this ThM movement changes the position of HD2 (small light-gray arrow) and brings out the second step: the rotation (curved light-gray arrow) of the DRD and HD1 of StXPB to shift HD1 (and RED motif) two bases along the 3′-overhang strand toward the duplex (see Insertion). Insertion: zoom-in view on the RED motif shifting along the 3′-overhang strand DNA. The curved blue arrow indicates the rotation. The shift of the RED motif from the DNA-free conformation (gray) to the DNA-bound conformation (green) is measured as the distance (11.5 Å, dash line) between the two positions of the RED motif residue D206. For comparison, the distance (10.8 Å, dash line) between two nucleotides (C13PO1 and T15PO1) is also shown.

In order to identify protein conformational changes induced by DNA binding, we also determined the crystal structure of the StXPB–Bax1^ΔC^ heterodimer at 2.96 Å resolution (see Supplementary Table S1 for statistics of data collection and structure refinement). In the StXPB–Bax1^ΔC^ heterodimeric structure, the ATP binding site of XPB contains a bound phosphate ion and the position of this phosphate ion is similar to that of the β-phosphate group of the ADP in the ADP-bound UvrB (47) (PDB entry: 2D7D, Supplementary Figure S3), an SF2 DNA helicase involved in bacterial NER. Therefore, this heterodimeric structure likely reflects StXPB in the (ADP + phosphate)-bound or ATP-bound conformation while the ternary structure presents StXPB in the ATP-free conformation. When the StXPB-Bax1^ΔC^ heterodimer and the ternary complex are aligned on Bax1^ΔC^, StXPB has substantial changes in domain orientation while Bax1^ΔC^ shows only local changes in the NTD caused by the movement of HD2 of StXPB: the ThM motif clamps down to intrude between the two arms at the junctions and the HD1 (and the N-terminal StXPB) rotates toward the dsDNA at the junction (Figure 4A). These domain re-arrangements in StXPB could be simply explained as a sequential two-step action induced by the forked DNA substrate and ATP binding/hydrolysis for StXPB to unwind DNA at the fork (Figure 4B). First, the initial DNA binding puts the forked DNA in the groove between the HD1 and HD2 of StXPB, ATP binding/hydrolysis allows the ThM motif to clamp down onto the ds–ss junction by intruding between the two ssDNA arms and gripping the 3′-overhang; this ThM movement changes the position of the HD2 since ThM is rigidly connected with HD2 (Figure 4B), which pushes HD1 and DRD to rotate toward the DNA duplex in order to maintain the forked DNA in the groove between HD1 and HD2. This second rotation shifts the HD1 of StXPB ∼11.5 Å from the 3′-ss tail into the duplex, equivalent to 2 bps (∼10.8 Å apart along the phosphate backbone) 3′ to 5′ forward movement along the 3′-overhang strand (Figure 4B, insertion), suggesting XPB could unwind two base pairs of dsDNA upon ATP binding and hydrolysis.

### StXPB has enhanced affinity for dsDNA with a small mismatched bubble

To test if the ThM clamping down into the DNA fork could enhance the interactions of StXPB with DNA distortion usually caused by NER lesions, we applied EMSA to compare the affinities of StXPB binding to normal dsDNA and dsDNA substrates with small mismatched bubbles ranging from 2-nt to 6-nt (Figure 3D). StXPB formed weak and unstable complexes with a 32-bp dsDNA substrate (smear in lane 2) and the bubble-2 (dsDNA with a 2-nt mismatched bubble) substrate (smear in lane 4). The interactions of StXPB with bubble DNA substrates increased when the size of the mismatched bubble increases from 2-nt to 5-nt (Figure 3D) and a distinguished band of the StXPB-DNA complex indicates that StXPB formed a stable complex with the bubble-4, bubble-5 and bubble-6 substrate (lane 8, 10, and 12 in Figure 3D). At high StXPB:DNA ratio, StXPB even formed a stable complex with the bubble-3 substrate (lane 4 in Figure 3E). Substitution of the RED motif with alanine residues (AAA) significantly reduced the interactions of StXPB with the bubble-3 (lanes 5–7 in Figure 3E) and bubble-5 substrates (lane 4 and 5 in Figure 3F). Deletion of the tip of the ThM motif (ΔThM1) almost eliminated the interactions of StXPB with the bubble-3 (lanes 8–10 in Figure 3E) and bubble-5 substrates (lanes 8 and 9 in Figure 3F). These results indicate that both the RED and ThM motifs are important for StXPB binding to distorted DNA, possibly playing a role in archaeal DNA damage recognition. To our surprise, substitution of F278 with Ala (F278A) did not show noticeable effects on the interaction of StXPB with the bubble-5 substrate (lanes 6 and 7 in Figure 3F). Furthermore, we analyzed the roles of these motifs in the ATPase activity of StXPB (Table 1). Mutations in the RED and ThM motifs including F278A significantly reduced the ATPase activity in the presence and absence of the bubble-5 DNA substrate and Bax1. These results indicate the importance of these motifs to the ATPase activity of StXPB in the order from the most important to the least important: the ThM motif (ΔThM2) > the RED motif > the ThM tip (ΔThM1) > residue F278. However, in the presence of the forked DNA substrate (Table 1), substitution of F278 with Ala has a much more severe effect (ATPase activity reduced to 47%) than the substitution of the RED motif with AAA does (ATPase activity reduced to 67%). These results together indicate that the RED and ThM motifs are important for bubble recognition and DNA unwinding at the fork while residue F278 is more important for DNA unwinding at the fork, consistent with its role as the wedge to break the base pairing at the fork (Figure 2).

**Table 1.** ATPase activities of StXPB variants in the presence and absence of Bax1 and DNA substrates

StXPB Variant	Alone	+StBax1	+StBax1 +Bubble-5 DNA	+StBax1 +forked DNA
**WT**	4.22 ± 0.22 (100%)^a^	12.05 ± 0.45 (100%) 2.9x^b^	53.99 ± 1.81 (100%) 12.8x^b^	86.74 ± 3.81 (100%) 20.6x^b^
**RED/AAA**	1.27 ± 0.18 (30%)	3.87 ± 0.29 (32%)	10.95 ± 0.92 (20%)	58.08 ± 1.14 (67%)
**F278A**	3.01 ± 0.04 (71%)	9.94 ± 0.26 (82%)	13.78 ± 0.19 (26%)	40.81 ± 0.94 (47%)
**ΔThM1**	2.07 ± 0.14 (49%)	7.02 ± 0.23 (58%)	12.05 ± 0.39 (22%)	31.52 ± 1.98 (36%)
**ΔThM2**	N.A.	2.48 ± 0.32 (21%)	3.71 ± 0.23 (6%)	9.84 ± 0.30 (11%)

^a^Numbers in the parenthesis represent the relative ATPase activities in the same column.

^b^ATPase activity enhancement over StXPB (WT).

The ATPase activities were obtained from at least three replicated experiments as described in the Materials and Methods.

ATPase activity unit: uM ATP hydrolyzed per uM protein per minute.

RED/AAA: StXPB mutant with R205A/D206A/D207A substitutions.

ΔThM1: StXPB mutant with deletion of residues 270–280.

ΔThM2: StXPB mutant with deletion of residues 258–299.

Bubble-5 DNA: Forked DNA:

5′- TAGTCACAGCTGA TTTTT CTCTGCTCCATAGT -3′ 5′-TAGTCACAGCTGATTGCGCTCTGCTCCATAGT-3′

3′-ATCAGTGTCGACTTTTTTGAGACGAGGTATCA-5′ 3′-ATCAGTGTCGACTAACGCGAGAGCTTCATAGT-5′

### Comparison to the cryo-EM structure of human TFIIH core complexed with XPA and a forked DNA

**Figure 5.**
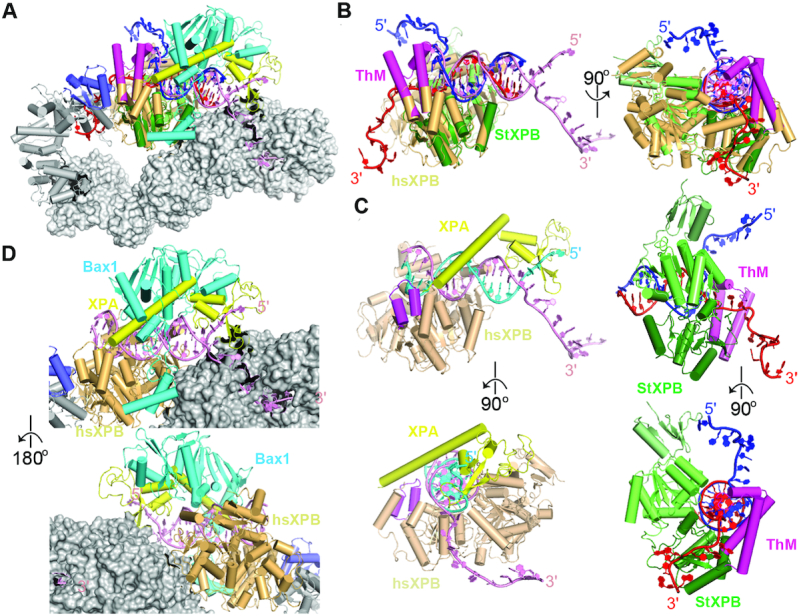
Structure comparison of the StXPB–Bax1^ΔC^–DNA ternary complex and the cryo-EM structure of the TFIIH–XPA–DNA complex. (**A**) Superimposition of StXPB in the StXPB–Bax1^ΔC^-forked DNA ternary complex with human XPB in the core TFIIH–XPA–DNA cryo-EM structure (PDB entry: 6RO4). Human XPB, p52, p8 and XPA are shown in light orange, gray, slate and yellow ribbons, respectively. The rest of the TFIIH core is shown in gray surfaces. Forked DNA in cryo-EM structure is shown in pink ribbons while the two DNA strands of the archaeal ternary structure are shown as red and blue ribbons, respectively. (**B**) Orthogonal views (left and right) of DNA–protein interactions for StXPB and human XPB as in (A) with other proteins omitted. (**C**) Orthogonal views (top and bottom) of the human XPB–XPA–DNA subcomplex (left) and the StXPB–DNA subcomplex (right). The two strands of DNA from the cryo-EM structure are highlighted in pink and cyan, respectively, for separation. The ThM motif of human XPB is highlighted in magenta. (**D**) Bax1 fits nicely with XPA at the DNA junction. Zoom-in of the front (top) and back (bottom) views as in (A) with both StXPB and the forked DNA omitted for simplicity.

XPB is conserved from archaea to human even though there is no TFIIH-like transcription/DNA repair factor in archaea. When our ternary complex is superimposed with the TFIIH-XPA-DNA cryo-EM structure (PDB entry: 6RO4) (31), a repair intermediate in human NER, over the HD2 domains of StXPB and human XPB (Figure 5A), not only are StXPB and human XPB aligned very well with both in the same closed conformation, but also the duplex regions of both DNA substrates in these two structures are surprisingly well matched (Figure 5B), sitting in the upper section of the groove formed between the two RecA-like (HD1, HD2) domains, indicating StXPB and human XPB interact with dsDNA in the same way as a dsDNA translocase. However, the two forked DNA substrates in our ternary complex and the cryo-EM structure point to the opposite directions (Figure 5A and B). In addition, human XPB is positioned about 5 bps away from the ds-ss junction while StXPB is right at the junction (Figure 5C), suggesting human XPB is more of translocase in the context of TFIIH. This is consistent with the fact that human XPB has a much shorter ThM motif (Supplementary Figure S4) and cannot clamp on the forked DNA like StXPB, as showing by the EMSA that human XPB forms unstable complex with the forked DNA substrate (Figure 3A). Interestingly, XPA seems to clamp on the forked DNA with a hook like the long ThM motif of StXPB (Figure 5C). The hook at the fork by XPA and the interactions of XPB at the duplex DNA complement each other and therefore enhance the overall protein-DNA interactions to form a stable ternary complex of human XPB-XPA with the forked DNA, strongly supporting our EMSA results (Figure 3A) and the previous observation that XPA can activate DNA unwinding by the TFIIH core (48). However, XPA grips the 5′-ss arm instead of the 3′-ss arm, which is bound by XPD in the cryo-EM structure (Figure 5D). In the StXPB–Bax1^ΔC^–DNA structure, StXPB grips the 3′-ss arm while Bax1 stabilizes the 5′-ss arm (Figure 1D). Remarkably, the nuclease StBax1 fits nicely with XPA together at the forked DNA in the cryo-EM structure (Figure 5D), suggesting that nuclease XPF or XPG could bind similarly like Bax1 to the junction with XPA and XPB (TFIIH core) for damage incision during eukaryotic NER.

## DISCUSSION

Conventional DNA helicases unwind DNA by loading to the ssDNA overhang of dsDNA and then translocating on this strand with cycles of ATP binding and hydrolysis to ‘unzip’ the dsDNA. However, XPB is believed to be an unconventional DNA helicase principally because XPB translocates along dsDNA instead of ssDNA (this feature makes the conventional helicase assay not applicable to detect DNA unwinding by XPB), but it is not clear how XPB unwinds duplex DNA as a translocase. Our structural and biochemical studies have uncovered that archaeal XPB homologs recognize the ds–ss DNA junction by interacting simultaneously with a short 3′-overhang and the DNA duplex immediately adjacent to the junction, and provide new insights to the unconventional DNA unwinding by XPB. Disruption of either the key RED or ThM motif impaired StXPB’s ability to interact with DNA, supporting that our DNA-bound ternary structure captures the state of repair bubble extension by the XPB-Bax1 machinery. Due to its shortened ThM motif, human XPB is more a translocase than a helicase, but XPA may complement this shortage and enhance its helicase activity for the DNA unwinding as the recent cryo-EM structure revealed that XPA has a hook clamping on the DNA fork and interacts with XPB simultaneously (31).

A typical NER DNA damage usually induces local melting of DNA. In eukaryotic NER, the XPC–HR23B complex firstly recognizes the lesion site. The β-hairpin of XPC that inserts into the double helix and flips out two base pairs (on the opposite strand of the damage) (16) is very similar to the ThM tip of archaeal XPB which also intrudes between two strands of the forked DNA. Since there is no XPC homologs existing in archaea, it is possible that archaeal XPB may also play a role in damage recognition (Supplementary Figure S5). The CPD-containing DNA fits nicely in the crystal structure of the StXPB-Bax1 heterodimer, likely reflecting the initial binding of XPB to the damage site (Supplementary Figure S5). Upon initial damaged DNA binding, XPB holds the dsDNA between the two RecA-like domains (HD1, HD2) with the ThM motif clamping at the lesion site. When the ThM motif clamps down, the tip of the ThM motif fits well into the void space created by the CPD and flipping out of the two bases on the other DNA strand (Supplementary Figure S5C), leading to the enhanced affinity of XPB binding to UV-damaged DNA over normal DNA, which would prevent the ThM motif from clamping down without melting the dsDNA. This is consistent with our EMSA results showing that StXPB forms a weak and unstable complex with dsDNA substrate but forms a stable complex with substrates containing a small bubble (Figure 3D). Because the ThM of XPB and Bax1 hold different strands of the melted DNA, this would further split apart the two DNA strands to create the initial repair bubble, which is then extended by the XPB-Bax1 machinery through ATP binding and hydrolysis. As described above, StXPB could unwind 2 bps per ATP binding and hydrolysis cycle (Figure 4B).

Interestingly, in the crystal structure of the StXPB–Bax1^ΔC^-forked DNA complex, the DNA is kept away from the nuclease domain of Bax1, therefore potentially preventing DNA incision by Bax1 until the DNA bubble is big enough for DNA repair. It is not yet known how the bubble size is determined during NER for any species. When our ternary complex is docked onto the TFIIH–XPA–DNA cryo-EM structure (PDB entry: 6RO4) (31) with StXPB superimposed with the human XPB, both the forked DNA and Bax1 fit nicely on the surface of the TFIIH core complex (Figure 5A and B). Our results suggest that XPG, like Bax1 associated with XPB, is kept away from the unwinding fork by XPB at the damage so that XPG does not incise the forked DNA prematurely.

## DATA AVAILABILITY

Atomic coordinates and structural factors for the structures described in this paper have been deposited in the Protein Data Bank under access codes 6P4W (StXPB–Bax1^ΔC^ heterodimer) and 6P4F (StXPB–Bax1^ΔC^–DNA ternary complex).

## Supplementary Material

gkaa801_Supplemental_File
