# Supplementary material for: Structural basis of the XPB helicase–Bax1 nuclease complex interacting with the repair bubble DNA
Source: Nucleic Acids Res. 2020 Sep 28;48(20):11695–705. doi: 10.1093/nar/gkaa801 (PMC7672443; doi:10.1093/nar/gkaa801)
Supplement: gkaa801_Supplemental_File [file gkaa801_supplemental_file.pdf]

Structural basis of the XPB helicase-Bax1 nuclease complex interacting with the repair bubble DNA

Feng He, Kevin DuPrez, Eduardo Hilario, Zhenhang Chen and Li Fan*

Department of Biochemistry, University of California, Riverside, CA 92521, USA..

*Correspondence and requests for materials should be addressed to L.F. (email: lifan@ucr.edu.)

SUPPLEMENTARY DATA

Figure S1. The StXPB-Bax1$^{\Delta C}$ complex interacts with DNA in the same way as the StXPB-Bax1 complex. Top: Sequence of the forked DNA substrate used for EMSA. Bottom: EMSA results. C, control reaction of the DNA substrate alone. The molar ratio of protein:DNA (0.5, 1.0, 1.5, and 2.0) for each reaction is indicated on the top of the gel. Experiments were repeated twice with consistent results.

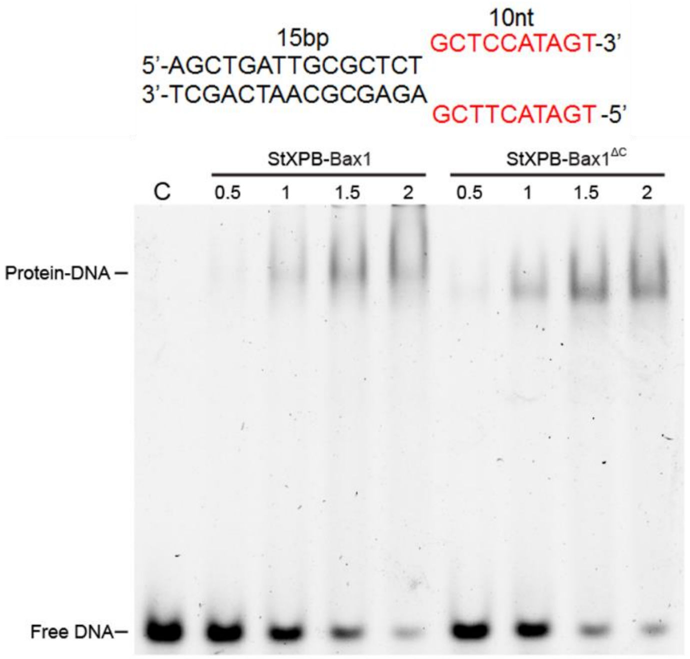

Figure S2. Electron density (Fo-Fc) map of the key residues highlighted in Figure 2. **(A)** The same view as in Figure 2B (upper). The electron density (Fo-Fc) map for residue F278 of StXPB and C13$_a$, C13$_b$ of the forked DNA is contoured at 2σ level in yellow. **(B)** The same view as in Figure 2B (lower). The electron density (Fo-Fc) map for residues R258 and W298 of StXPB as well as bases C14$_a$ and T15$_a$ of the forked DNA is contoured at 2σ level in yellow. **(C)** The same view as in Figure 2C. The electron density (Fo-Fc) map for the RED motif residues R205, D206, D207 and bases A11$_a$, C14$_a$, C13$_a$ of the forked DNA is contoured at 2σ level in yellow.

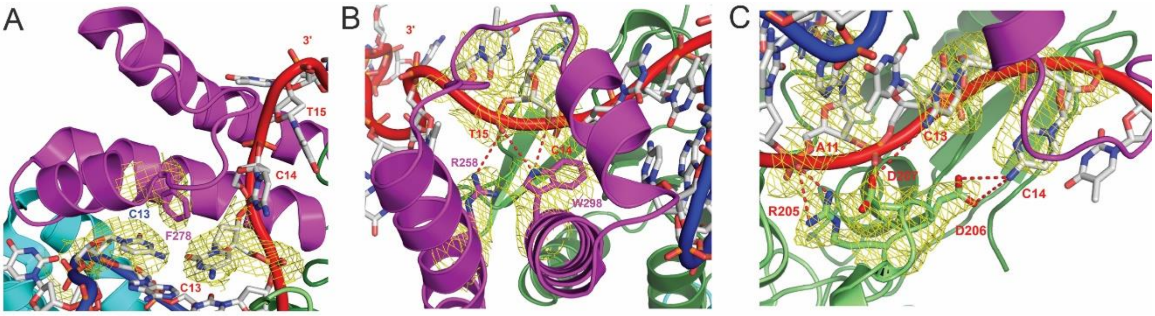

Figure S3. The ATP binding site of StXPB in the StXPB-Bax1$^{\Delta C}$ structure. (A) StXPB is colored in green with the bound phosphate ion colored in red and orange. The residues possibly involved in forming the ATP-binding pocket are shown in sticks. (B) Overlay of key residues at the ATP-binding sites in StXPB (green) and UvrB (PDB entry: 2D7D, wheat)(1). The bound phosphate ion in StXPB-Bax1$^{\Delta C}$ structure is colored as in (A) and the bound ADP in the UvrB structure is colored in wheat.

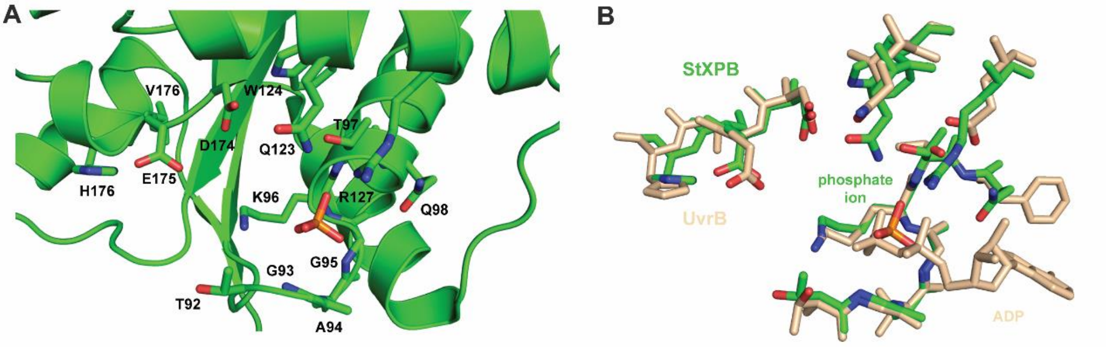

Figure S4. Sequence and structural alignment of StXPB and human XPB. Alignment was performed with PROMALS3D (2) and depicted using ESPript 3.0 server (3). Secondary structure elements for StXPB (top) and human XPB (bottom) are numbered and represented according to the PDB files of the StXPB-Bax1$^{\Delta C}$-DNA (PDB entry: 6P4F) and core TFIIH-XPA-DNA (PDB entry: 6RO4)(4) structures. The secondary elements of the ThM motifs for both human XPB and StXPB are highlighted by magenta boxes.

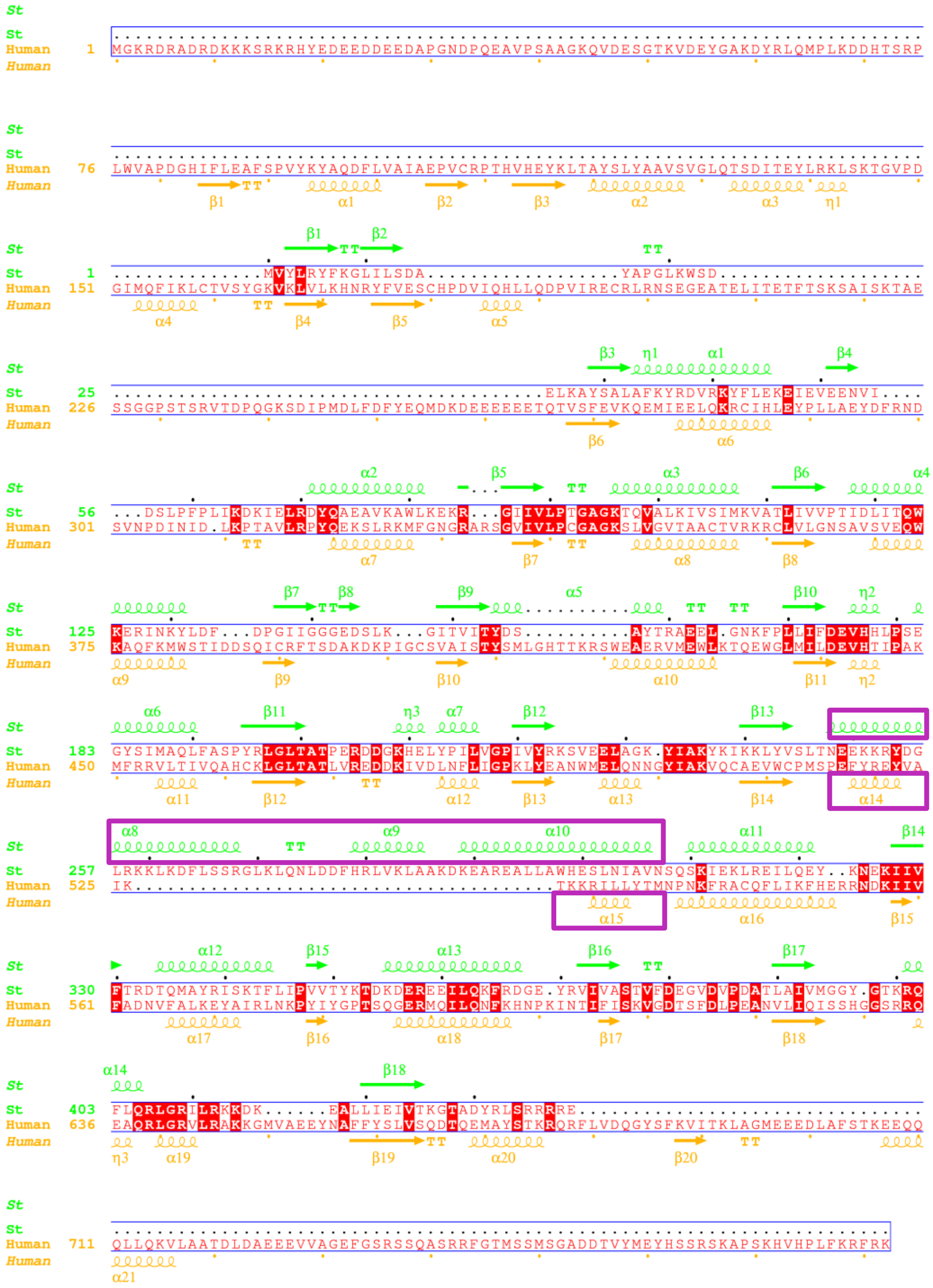

Figure S5. Model of StXPB binding to the damage site. (**A**) CPD-containing DNA (orange) from the Rad4-Rad24-DNA complex (PDB entry: 2QSG)(5) is superimposed with the forked DNA in the ternary complex over the dsDNA region. Bax1 is shown in cyan ribbons with the Cas2-like domain highlighted in yellow and the nuclease motifs in red. (**B**) Two views of the ternary complex with the forked DNA replaced by the CPD-containing DNA from (A). Domains of StXPB are colored differently. (**C**) Zoom-in view of the ThM tip clamping into the void space created by the lesion CPD.

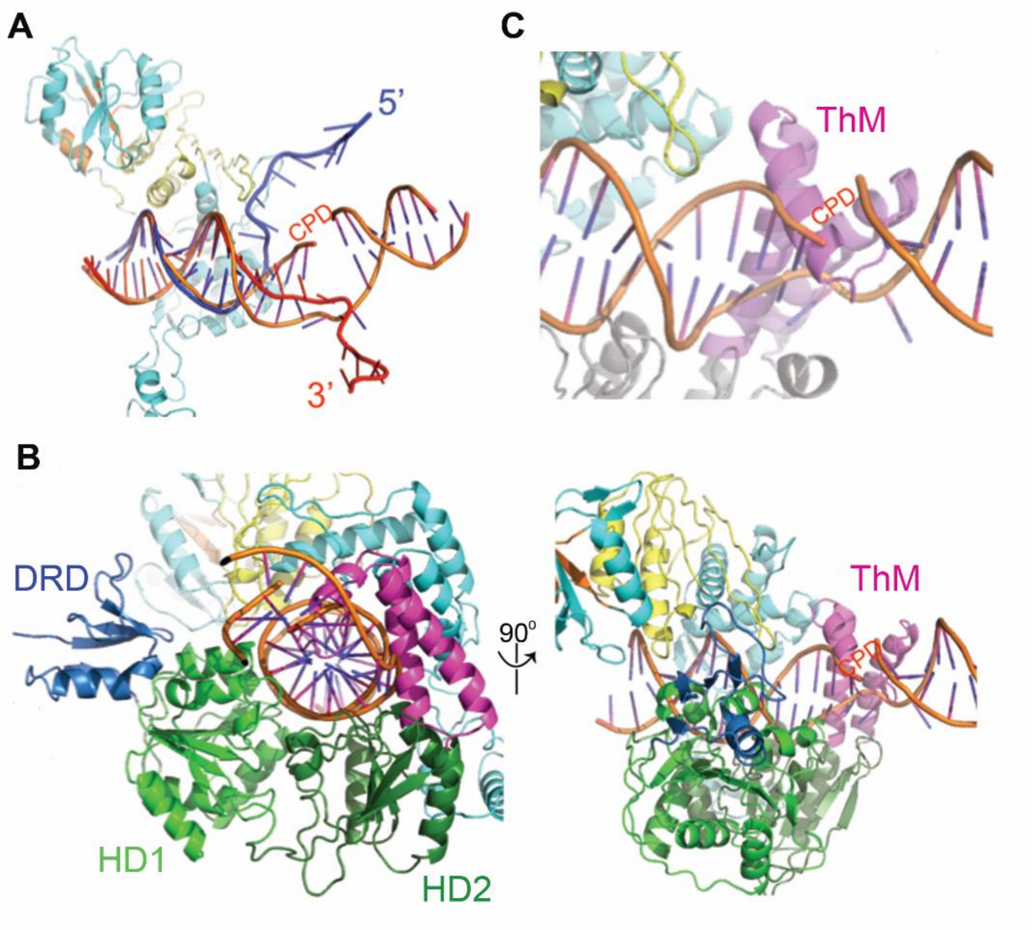

Table S1. Statistics for X-ray diffraction data collection and structural refinement.

Structure	StXPB:Bax1$^{\Delta C}$	StXPB:Bax1$^{\Delta C}$-DNA
PDB ID	6P4W	6P4F
**Data collection**		
Space group	P 1	C1 2 1
Cell dimensions: a, b, c (Å)	56.42, 101.37, 114.48	214.69, 92.15, 172.13
α, β, γ (°)	83.09, 81.15, 90.17	90.00, 132.24, 90.00
Resolution (Å)	29.28 – 2.96 (3.00 – 2.96)	39.86 – 3.55 (3.74 – 3.55)
R_{pim}	0.061 (0.529)	0.148 (0.973)
$I/\sigma I$	8.9 (1.0)	4.9 (1.3)
Completeness (%)	99.1 (98.3)	94.1 (94.3)
Multiplicity	3.5 (3.3)	3.0 (3.0)
CC1/2 (%)	98.8 (63.2)	97.7 (30.5)
**Refinement**		
Resolution (Å)	29.28 – 2.96 (3.06-2.96)	39.74 – 3.55 (3.68-3.55)
No. reflections	48672	28335
R_{work} / R_{free}^{*}	18.30 / 23.34	25.02 / 27.16
Number of atoms	13068	14209
Protein	12930	12310
Ligands	42	1899
Water	96	0
Ramachandran favored	95.71%	91.80%
Ramachandran allowed	4.29%	8.20%
Ramachandran outliers	0.00%	0.00%
R.m.s.d Bond length	0.011 Å	0.004 Å
R.m.s.d Bond angles	1.43°	0.73°
Fo, Fc correlation	0.95	0.91
Anisotropy	0.040	0.128
Averaged B factor	80.22 Å^2	108.8 Å^2
MolProbity score	1.86	2.05

Values in parenthesis are for the highest resolution shell. *5% data was used for R_{free}.

SUPPLEMENTARY REFERENCES

1. Eryilmaz, J., Ceschini, S., Ryan, J., Geddes, S., Waters, T.R. and Barrett, T.E. (2006) Structural insights into the cryptic DNA-dependent ATPase activity of UvrB. *J Mol Biol*, **357**, 62-72.

2. Pei, J., Kim, B.H. and Grishin, N.V. (2008) PROMALS3D: a tool for multiple protein sequence and structure alignments. *Nucleic Acids Res*, **36**, 2295-2300.

3. Robert, X. and Gouet, P. (2014) Deciphering key features in protein structures with the new ENDscript server. *Nucleic Acids Res*, **42**, W320-324.

4. Kokic, G., Chernev, A., Tegunov, D., Dienemann, C., Urlaub, H. and Cramer, P. (2019) Structural basis of TFIIH activation for nucleotide excision repair. *Nature communications*, **10**, 2885-2904.

5. Min, J.H. and Pavletich, N.P. (2007) Recognition of DNA damage by the Rad4 nucleotide excision repair protein. *Nature*, **449**, 570-575.
